# Association Between ABCB1 (MDR1) Gene Polymorphism and Unresponsiveness Combined Therapy in Chronic Hepatitis C virus

**DOI:** 10.5812/hepatmon.7522

**Published:** 2013-04-13

**Authors:** Meryem Timucin, Hakan Alagozlu, Semra Ozdemir, Ozturk Ozdemir

**Affiliations:** 1 Department of Gastroenterology, Faculty of Medicine, Cumhuriyet University, Sivas, Turkey; 2 Department of Nuclear Medicine, Faculty of Medicine, Canakkale Onsekiz Mart University, Canakkale, Turkey; 3 Department of Medical Genetics, Faculty of Medicine, Cumhuriyet University, Sivas, Turkey; 4 Department of Medical Genetics, Faculty of Medicine, Canakkale Onsekiz Mart University, Canakkale, Turkey

**Keywords:** Hepatitis C, Data Collection, P Glycoprotein

## Abstract

**Background:**

To treat viral infection of chronic hepatitis C (CHC) is a main strategy to prevent progression of liver disease, and cancer. Some patients with CHC have failed to respond to the common antiviral therapy in different populations.

**Objectives:**

In the current study it was aimed to find out the possible role of multiple drug resistance gene1 (MDR1) in non-responder patients with CHC infection in Turkish population.

**Patients and Methods:**

Peripheral blood-EDTA samples were used for total genomic DNA isolation. In total of 55 patients with chronic hepatitis C and positive results for genotype 1 [31 male (56.4%), 24 female (43.6%) and mean age-min-max; 56.9 ± 9.66 (39-71)]; 19 responder (34.5%), 21 non responder (38.2%), and 15 recurrence (27.3%) were included in the presented results. Functional MDR1 gene was genotyped by multiplex PCR-based reverse-hybridization Strip Assay method, and some samples were confirmed by direct sequencing.

**Results:**

Our results indicate that MDR1 gene polymorphism is strongly associated with non-responder patients and those with recurrent chronic hepatitis C during conventional drug therapy when compared to the responder patients. Homozygous of the TT genotype for MDR1 exon 26 polymorphism was at 2.0-fold higher risk of non-responder than patients with CC and CT.

**Conclusions:**

The homozygous MDR1 3435TT genotype which encodes the xenobiotic transporter P-glycoprotein may be associated with a poor antiviral response in HCV chronicity during conventional therapy, and large-scale studies are needed to validate this association.

## 1. Background

Positive sense single-stranded RNA virus, HCV is from the virus family of Flaviviridae, and causes to the blood-borne liver infection primarily in hepatocytes in human (1). Hepatitis C infection is accepted as a metabolic liver disease more than virus based infectious disease because of the high prevalence of metabolic alterations. The primary goal of medical treatment of HCV infection is to prevent the liver complications such as; progression of liver disease, and cancer. The peginterferon alfa, and ribavirin are the common medical drugs which are currently used in the HCV treatment. In some population nearly a half of patients failed to respond to an earlier version of antiviral therapy of HCV infection (1-3). Alternative treatment strategies are needed to cure nonresponder patients of HCV infection. Approximately, 180 million individuals are affected by acute or chronic viral hepatitis, and its prevalence is estimated to reach 2% of the world population (4); moreover, about 60% of these HCV carriers are also at the risk of HCV related several life threatening complications, and extrahepatic manifestations such as cryoglobulinemia (4-9). Concurrently, persistent chronic HCV infection more potent to developed cirrhosis, end stage of liver disease, and hepatocellular carcinoma is a result of hepatocellular necrosis among other hepatitis virus types (4, 10). HCV infection has been linked to severe chronic conditions such as; altered lipid metabolism (5, 10, 11), high prevalence of steatosis (11), fibrosis (12) increased insulin resistance (IR), (13-15), and type 2 diabetes mellitus (16). The single nucleotide polymorphism (SNP) in drug efflux genes such as drug transporter genes, peroxisomal genes, transferases are new highlights of current research. Although genomic heterogeneity of HCV virus affects the efficiency of the antiviral therapy, therapy response against HCV is also associated with host genetic profile (17). The recent experiments for understanding the link between association of different gene polymorphisms with drug pharmacokinetics and viral kinetics, is a promising tool for prediction of therapy response, and vital for “hard to cure” or “null-responder” patients. For instance, Peg-IFN-a plus Ribavirin therapy efficiency on chronic HCV patients is closely related to the miR196a-2 gene polymorphism (18). Another polymorphism based treatment response study indicates that the tumor necrosis factor-related apoptosis-inducing ligand receptor 1 (TNFRSF10A) gene rs4242392 SNP causes low-degree interferon-based response against HCV (19). Moreover, rs12979860 polymorphism, located upstream region of IL-28B interleukin, enhanced response rates in chronic hepatitis C infected patients treated with (PEG-INF) plus ribavirin (RBV) (20).

The multidrug resistance gene 1 (MDR1, adenosine triphosphate-binding cassette transporter ABCB1, P-glycoprotein) encodes 170 kDa, glycosylated efflux transporter membrane protein which plays a crucial role in protecting cells from xenobiotics, chemicals, and drugs (21, 22) such as; immunosuppressant (23), antidepressant (24), Ca^+2^ channel blockers (25), chemotherapeutics (26), and antiviral therapeutics (27). This gene is mainly expressed in apical sides of barrier organs like intestinal, renal, hepatic epithelial cells or brain endothelial cells, thus polymorphism on MDR1 gene affects pharmacokinetics, and pharmacodynamic of a broad spectrum of substances and xenobiotics. The MDR1 gene has a total of 50 different polymorphic alleles, and 20 of them has silent feature (28).

## 2. Objectives

In the current case-control study it was aimed to find out the possible association between MDR1 polymorphism and gene based unresponsiveness and HCV infection.

## 3. Patients and Methods

### 3.1. Patients and Clinical Diagnosis

The current study was performed in Departments of Gastroenterology and Medical Genetics of Cumhuriyet University Hospital. All applications were approved by the local ethics committee of Cumhuriyet University (Ref No: 2009-06/22). Informed consent was obtained from all of the subjects. Blood samples with EDTA from patients with chronic hepatitis C, and positive results for genotype 1 who underwent medical treatment to cure the viral infection were used for genotyping in the current case-control study. The viral load quantitation was established by the single-tube RT-PCR technique from peripheral blood samples for each patient according to the manufacturer’s instructions. The histology activity index (HAI) was evaluated by Knodell's scoring system which is the combined scores for necrosis, inflammation, and fibrosis for all groups. In a total of 55 patients with positive results for HCV [31 male (56.4%), 24 female (43.6%) and mean age-min-max; 56.9 ± 9.66 (39-71)]; 19 responder (34.5%), 21 nonresponder (38.2%), and 15 recurrence (27.3%) were included, genotyped for the MDR1 gene polymorphism and compared with the other groups in the presented results. Other causes of chronic hepatitis except for HCV were excluded by appropriate serological tests, and liver histology.

### 3.2. Genotyping and Mutation Analysis

Total genomic DNA was extracted from 100 μL peripheral blood samples from patients by the Invitek kit extraction technique (Invitek, Invisorb spin blood, Germany), and stored at −20°C until genetic analysis was performed. The mutation analysis of target MDR1 gene was performed by Strip Assay technique (Vienna Lab, Strip Assay GmbH, Austria) which is based on the reverse-hybridization principle automatically. MDR1 gene was simultaneously amplified and biotin-labeled in a single multiplex amplification reaction (Viennalab, Strip Assay GmbH, Austria). PCR for MDR1 gene was performed in a Perkin Elmer 9600, (Singapore), and the protocol consisted of an initial melting step of 2 min at 95°C; followed by 35 cycles of 15s at 95°C, 30s at 56°C, and 30s at 72°C; and a final elongation step of 3 min at 72°C (29).

### 3.3. Statistical Analysis

The current results from different HCV positive group patients were given as the mean (standard deviation [SD]). The software SPSS for Windows version 12.0 was used to perform statistical analysis. The Mann-Whitney U and *X*^*2*^ tests were used to analyze differences among the three patient groups.

## 4. Results

We studied the association between C3435T polymorphisms of MDR1 gene and its correlation with drug resistance against “nonresponder” patients with HCV. In a total of 55 seropositive genotype 1 patients with HCV; 19 responder [8 female (42.1%), and 11 male (57.9%)], (cases that were found to have seronegative HCV-RNA and normal ALT values after 6 months treatment), 21 nonresponder [10 female (47.6%), and 11 male (52.4%)], (cases that were found to have seropositive HCV-after 6 months treatment and increased ALT and fibrosis values), and 15 recurrence patients [6 female (40.0%) and 9 male(60.0%)], (cases that were found to have seronegative HCV-RNA after 6 months treatment but HCV-RNA levels were also raised to untreated level after second 6 months period) were examined in the presented results. The mean HCV-RNA loads value were > 50 IU/ml for the current patients with HCV at the beginning of the treatment, and all patients with negative results for serum HAV, HBV, HDV, and HIV were excluded. The current cohort includes 24 female (43.6%), and 31 male (56.4%) patients with HCV and positive results for genotype 1 (Table 1). The viral load was 2.7×106 ± 5.45×105 for responder, 4.7×106 ± 1.01×106 for nonresponder, and 5.7×106 ± 1.02×106 for recurrence group before treatment respectively (Table 2). After combined treatment with alpha 2a-interferon, and ribavirin the viral load has changed to zero (0) for responder, 4.5×106 ± 1.30×106 for nonresponder, and 4.6×106 ± 7.40×105 for recurrence group respectively (Table 2).

**Table 1.** Some Clinical Characteristics for the Current Responder, Nonresponder, and Recurrent Patients

Characteristics	Patients (n = 55)			P value
	Responders (n = 19)	Non responders (n = 21)	Recurrence (n = 15)	
**Age, y, Mean ± SD (Min-Max)**		56.9 ± 9.66 (39-71)		-
**Gender, No. (%)**				-
Female		24 (43.6)		-
Male		31 (56.4)		-
**Fibrosis Score, Mean ± SD (0-4)**	2.38 ± 2.09	2.25 ± 1.83	2.40 ± 2.11	0.999
**ALT, Mean ± SD, U/L**	32.84 ± 28.10	62.57 ± 44.39	60.20 ± 50.70	0.017
**HAI Score, Mean ± SD (0-10)**	5.66 ± 1.53	5.65 ± 1.22	5.10 ± 2.60	0.396

**Table 2.** Shows Mean Viral Load (IU/ml) Before, and After Treatment for all Groups

Patients	Viral Load, mean ± SD, IU/ml	
	Before Treatment	After Treatment
**Responder**	2.7×10^6^± 5.45×10^5^	0
**Nonresponder**	4.7×10^6^± 1.01×10^6^	4.5×10^6^± 1.30×10^6^
**Recurrence**	5.7×10^6^± 1.02×10^6^	4.6×10^6^± 7.40×10^5^

All groups have nearly the same fibrosis and HAI profiles but the ALT values were significantly different in responder group when compared to the nonresponder, and recurrence groups (Table 1), (P = 0.017). Six of 19 (31.6%) responder patients were in homozygous CC genotype (wild type), and 13 (68.4%) patients were in heterozygous CT genotype (Table 3). The homozygous mutated TT genotype was not detected in the current responder cohort (Table 3). Three of 21 (14.2%) nonresponder patients were in homozygous CC genotype, 9 (42.9%) patients were in heterozygous CT, and 9 (42.9%) patients of nonresponder were in homozygous mutated TT genotype (Table 3). Two of 15 (13.3%) recurrence patients were in homozygous CC genotype, and 13 (86.7%) patients were in heterozygous CT genotypes. The homozygous mutated TT genotype was also not detected in the current recurrence cohort (Table 3). When individuals at each group were compared according to the prevalence for the MDR1 gene mutation profiles, the difference among the groups was considered as logical statistically significant (P < 0.05), (*X*^2^:19.26), (Tables 2 and 3). The C and T allele frequency of 3435 C > T SNP for all groups were; 0.658 for C, and 0.342 for T allele in responder group, 0.357 for C, and 0.693 for T allele in nonresponder group, and 0.567 for C, and 0.433 for T allele in current recurrence group (Table 3). Increased mutated T allele frequency for MDR1 exon 26 genes was detected in the presented nonresponder cohort. The difference for T allele was statistically significant when compared to the responder group (P = 0.001) (Table 3).

**Table 3.** Mutation Distribution, Genotype, and Allele Frequency of the MDR1 Gene [P-Glycoprotein, ATP-Binding Cassette (ABC) Superfamily of Membrane Transporters] 3435 C > T SNP in the Current Responder, Non responder and Recurrent HCV Positive Groups a

Patients (n = 55)	Genotype CC, No. (%)	Genotype CT, No. (%)	Genotype TT, No. (%)	Allele Frequency C	Allele Frequency T
**Responder (n = 19)**	6 (31.6)	13 (68.4)	0	0.658	0.342
**Nonresponder (n = 21)**	3 (14.2)	9 (42.9)	9 ( 42.9)^b^	0.357	0.693
**Recurrence (n = 15)**	2 (13.3)	13 (86.7)	0	0.567	0.433

^a^P = 0.001; *X*^2^ = 19.26; Gene, MDR1 (ABCB1); Exon, 26; SNP, C > T, Transition; Codon, 3435

^b^P < 0.05 significant

## 5. Discussion

Hepatocellular carcinoma (HCC) is one of the most common malignant cancers which is closely associated with chronic infection by the hepatitis B (HBV) and C (HCV) viruses in various populations (30-33). High rate of mortality, cirrhosis risk development, and HCC may occur in the long time unresponsiveness treatment of patients with HCV infection (31, 34-36). Increased MDR1 expression was reported in tumoral tissues of HCC when compared to the nontumoral samples (37). Rose et al., have claimed that up-regulation of such genes as; MDR1, MRP1, and MRP3 are strongly associated with hepatocytes in severe human liver disease (38). Funaoka et al., have claimed that the 70 and 91 amino acids substitution in HCV core region is a significant predictor of poor responses to peginterferon-plus-ribavirin therapy by detecting the lower HCV RNA in culture supernatant than the wild type subclones (39). They also claimed that the infected patients by mutated virus showed increased incidence of HCC. Viral drug unresponsiveness is sometimes based on viral and/or bacterial gene recombinations, and resistance to the wide range of drugs (29, 40-42) but in general it is based on genomic variation and/or mutations of host organisms genome, intracellular functions and causes different tissue cancers in human (43-47). As claimed by Hoofnagle et al., the racial difference (variation in host genome) takes crucial role in the response to antiviral therapy (48). These results suggest that racial differences in the response to antiviral therapy are due to greater unresponsiveness to intracellular actions of interferon in African, American individuals after peginterferon alfa-2a and ribavirin therapy without dose modification in HCV genotype 1 (48). Artini et al., have claimed that elevated serum 90K/MAC-2 BP levels are related to the degree of disease severity, infection period, and they are acceptable for the independent predictor for the failure to respond to alpha-interferon treatment in patients with CHC (49). We studied the association between C3435T polymorphisms of MDR1 gene, and its correlation with combined drug resistance in the nonresponders patients with positive results for HCV during conventional therapy. Three HCV seropositive groups (responder, nonresponder and recurrence) were compared in the presented results. The current results from twenty one seropositive HCV patients [10 male(47.6%) and 11 female(52.4%)] who were treated with combined alpha 2a-interferon, and ribavirin for 6 months showed strong association in the MDR1 SNP and drug unresponsiveness in patients with HCV. The mutation profile of MDR1 affects the response rates and clinical progress on the current cohort of patients with hepatitis C in Turkish population. All nonresponder patients have also increased ALT and fibrosis values after treatment period of 6 months. None of the current responder and recurrence group patients have TT genotype but a large amount of the nonresponder patients (42.9%) showed homozygous mutated TT genotype (Table 3). The presented results showed statistical significance for both TT genotype, and T allele frequency for nonresponder group (P < 0.05), (*X*^*2*^:19.26), (Tables 2 and 3). Increased mutated T allele frequency was detected in the nonresponder cohort when compared to the other two groups in Turkish population. According to the presented results, while higher rate of heterozygote C > T was seen for three responded groups to treatment, higher rate of homozygote was detected on patients with hepatitis C who are unresponsive to treatment. This shows that there is a strong association between MDR1 polymorphism and HCV unresponsiveness. The P-glycoprotein which is encoded by MDR1 effluxes many chemicals. The silent C3435T mutation in MDR1 gene causes decrease in MDR1 expression, and also in the amount of MDR1 receptors (23, 26, 28). Variation in exon 26 of MDR1 gene also shows significant differences for drug transport. Therefore it affects both response and therapeutic doses of multiple drugs (22-24, 29). The homozygous C alleles in C3435T SNP are considered to be responsible for resistance against the medicines in which the P-gp expression is high and against some materials. The TT genotype of the same SNP in exon 26 of MDR1 gene causes over expression of gene activity, and effluxes many chemicals. Some recent reports insist that the TT alleles failed to efflux chemicals role, and cause to some types of cancer such as; HCC (30-32), breast (47), NSCLC (46), prostate (43), and ovarian cancers in human (44, 45). In conclusion, the homozygous TT alleles in MDR1 gene showed strong association with drug unresponsiveness in the current nonresponder patients with HCV who were treated by combined alpha 2a-interferon, and ribavirin for six months. Results also showed that there is increased T allele frequency for MDR1 exon 26 genes in current nonresponder cohort in Turkish population. Results need to be supported by population based large–scale samples of representative nonresponder patients with HCV.
